# Application of the ICD-PM classification system to stillbirth in four sub-Saharan African countries

**DOI:** 10.1371/journal.pone.0215864

**Published:** 2019-05-09

**Authors:** Mamuda Aminu, Matthews Mathai, Nynke van den Broek

**Affiliations:** Centre for Maternal and Newborn Health, Liverpool School of Tropical Medicine, Liverpool, United Kingdom; Public Health Foundation of India, INDIA

## Abstract

**Objective:**

To identify the causes and categories of stillbirth using the Application of ICD-10 to Deaths during the Perinatal Period (ICD-PM).

**Methods:**

Prospective, observational study in 12 hospitals across Kenya, Malawi, Sierra Leone and Zimbabwe. Healthcare providers (HCPs) assigned cause of stillbirth following perinatal death audit. Cause of death was classified using the ICD-PM classification system.

**Findings:**

1267 stillbirths met the inclusion criteria. The stillbirth rate (per 1000 births) was 20.3 in Malawi (95% CI: 15.0–42.8), 34.7 in Zimbabwe (95% CI: 31.8–39.2), 38.8 in Kenya (95% CI: 33.9–43.3) and 118.1 in Sierra Leone (95% CI: 115.0–121.2). Of the included cases, 532 (42.0%) were antepartum deaths, 643 (50.7%) were intrapartum deaths and 92 cases (7.3%) could not be categorised by time of death. Overall, only 16% of stillbirths could be classified by fetal cause of death. Infection (A2 category) was the most commonly identified cause for antepartum stillbirths (8.6%). Acute intrapartum events (I3) accounted for the largest proportion of intrapartum deaths (31.3%). In contrast, for 76% of stillbirths, an associated maternal condition could be identified. The M1 category (complications of placenta, cord and membranes) was the most common category assigned for antepartum deaths (31.1%), while complications of labour and delivery (M3) accounted for the highest proportion of intrapartum deaths (38.4%). Overall, the proportion of cases for which no fetal or maternal cause could be identified was 32.6% for antepartum deaths, 8.1% for intrapartum deaths and 17.4% for cases with unknown time of death.

**Conclusion:**

Clinical care and documentation of this care require strengthening. Diagnostic protocols and guidelines should be introduced more widely to obtain better data on cause of death, especially antepartum stillbirths. Revision of ICD-PM should consider an additional category to help accommodate stillbirths with unknown time of death.

## Introduction

Stillbirth is a major public health problem, with an estimated 2.6 million deaths occurring annually worldwide, 98% of which occur in low- and middle-income countries (LMIC) [1]. Many LMIC have stillbirth rates above 30 per 1,000 births which is at least three times higher than in high-income countries.

To achieve the global target of reducing the stillbirth rate to 12 or fewer per 1,000 births in every country by the year 2030 [2], it is important that availability and quality of care during pregnancy and childbirth is improved and action is taken to tackle the major causes of stillbirth. Although the main causes of stillbirth in LMIC are considered to be intrapartum hypoxia, placental conditions and infections, the cause is unknown for a substantial proportion [3, 4].

Classification systems help to categorise cause of stillbirth into related groups to aid the development of strategies and programmes to address the underlying causes of and factors resulting in death. A variety of classification systems have been used for stillbirth [5], most of which show poor comparability [6], and, in the literature from LMIC, about two-thirds of stillbirths are consistently rated as unexplained or cause unknown [7]. Some of the systems cannot be recommended for classification of cause of stillbirth as they were not designed for this, and others are considered difficult to apply and have been reported to have very high inter-observer variability [6].

In 2016, the World Health Organisation adapted the existing International Classification of Diseases (ICD-10) for use in perinatal mortality—The WHO Application of ICD-10 to Deaths during the Perinatal Period [8]. The new ICD-PM classification system uses a layered approach to categorise perinatal mortality (including stillbirth) in the first instance by time of death (antepartum, intrapartum and neonatal deaths), and can then be used to further assign a fetal cause of death and/or contributing maternal conditions. It is hoped that this new classification system will facilitate more accurate and uniform reporting to enable comparison within and between settings. Although the ICD-PM has been shown to work well using data from the UK and South Africa [9, 10], there is limited data about its application in LMIC settings where the majority of stillbirths occur, and disease epidemiology may be different.

We conducted a study to identify the major causes of stillbirth in LMIC settings applying the new ICD-PM. In addition, we wished to identify potential areas for improving the ICD-PM classification system, if any.

## Methods

### Ethical approval

This study was approved by the Ethics Committee of the Liverpool School of Tropical Medicine (14.026) as well as by individual Ethics Committees in all the four study countries: Kenyatta National Hospital/University of Nairobi Ethics & Research Committee (KNH-ERC/A/398), Malawi College of Medicine Research & Ethics Committee (p.07/14/1601); Sierra Leone Ethics and Scientific Review Committee (09/10/2014 and 31/08/2015), and; the Medical Research Council of Zimbabwe (MRCZ/A/1895).

### Study sites

Participating hospitals were healthcare facilities designated to provide comprehensive emergency obstetric care located in Kenya (3 hospitals), Malawi (4 hospitals), Sierra Leone (2 hospitals) and Zimbabwe (3 hospitals) and involved in the Making it Happen (MiH) programme [11] which aimed to improve availability and quality of maternal and newborn health care in sub-Saharan Africa and Asia. In each participating district, the hospitals with the highest number of births were selected to participate in the programme including both at tertiary and secondary level. Even though all included facilities were designated to provide comprehensive emergency obstetric care, their capacity to deliver such services varied.

Quality improvement activities, including perinatal death audit and maternal death audit, were introduced in these hospitals through the MiH programme. In each healthcare facility, a team of between eight and twelve healthcare providers (midwives, nurses, doctors and clinical officers) involved in the provision of maternal and newborn care, were trained to conduct perinatal death audit. In this study, a stillbirth was defined as a baby born without any sign of life at 28 weeks of gestation or more, or with a birthweight of 1000g or more [12].

### Definitions

**Antepartum death**: Death before the onset of labour. In this study, this was defined as macerated stillbirths whose mothers arrived at the healthcare facility without fetal heart sound or where fetal heart rate was not documented.

**Intrapartum death:** Death after the onset of labour or during birth. This included stillbirths documented as fresh stillbirth and stillbirths whose mothers were admitted in labour with fetal heart sound present irrespective of physical appearance at time of birth.

**Unknown time of death:** For the purpose of this study, any case that could not be categorised as either an antepartum or intrapartum death was categorised as unknown time of death. An additional category was created for this group, and the letter U (representing unknown) was used to code these using the following sub-categories: congenital malformations, deformations and chromosomal abnormalities (U1), hypoxia and other acute events (U2), infection (U3), other specified disorder (U4), disorders related to fetal growth (U5), and death of unspecified cause (U6).

### Data collection

The total number of births, live births and stillbirths was obtained for each month during the study from existing healthcare facility registers (labour ward, discharge and theatre registers). On a monthly basis, healthcare providers in each of the participating hospitals reviewed all stillbirths that had occurred in the preceding month. Data was collected for births that occurred between January and July 2015, but this was extended to September 2015 in Sierra Leone due to lower number of cases than anticipated in the target hospitals during the Ebola virus epidemic. In Zimbabwe, however, the data collection ended in April 2015, when the sample size was reached.

Information was extracted and triangulated from different sources, including case records, handover notes and hospital registers, using a pre-designed data collection form to summarise case findings. Data collected included the date of birth, maternal sociodemographic characteristics, pregnancy details, obstetric and medical history, baby’s characteristics (sex, weight, physical appearance), documented cause of death and factors that might have contributed to the death. Other variables required for use as denominators in calculating rates (total births and total live births) were obtained from labour ward and theatre registers. No specific additional diagnostic screening was possible or had been conducted in participating hospitals.

### Sample size

All stillbirths were identified sequentially until a predetermined sample size of 279 per country was reached. With this sample size, if the proportion with a given cause of death was 24%, the margin of error would be 5% using the 95% confidence level. In each country, the sample to be achieved was divided between the hospitals based on the number of births expected in each hospital. For the purpose of this study, data collection was discontinued when the predetermined sample size in each country was reached.

### Assigning cause of death

The trained team of healthcare workers in each of the participating hospitals reviewed all stillbirths within one month of the death occurring and summarised information from patient records and hospital registers into a specially designed data collection tool (S1 File). For each case, they identified the most likely fetal cause of death as well as other contributing maternal conditions via consensus.

### Application of ICD-PM

Cases were categorised by time of death; antepartum, intrapartum stillbirth or unknown. The most likely fetal cause of stillbirth and/or the contributing maternal condition if known were classified as per the provisions of the ICD-PM classification system [8]. Antepartum deaths were further classified into the six ICD-PM sub-categories (A1 to A5, with A6 representing cause unknown), and; intrapartum deaths were classified into seven sub-categories (I1 to I6, with I7 representing unknown cases). The contributing maternal conditions were classified into five major categories (M1 to M4, with M5 representing the unknown cases) (Table 1) [8].

**Table 1:** ICD-PM categories with description and exemplar-specific causes.

Time of death	Category	Description	Examples
**Antepartum death**	A1	Congenital malformations and chromosomal abnormalities	Anencephaly, encephalocele, microcephaly, congenital hydrocephalus, spina bifida, etc.
	A2	Infection	Congenital syphilis, congenital malaria, congenital rubella syndrome, congenital TB, etc.
	A3	Antepartum hypoxia	Intrauterine hypoxia
	A4	Other specified antepartum disorder	Vasa previa, ruptured cord, twin-twin transfusion, Intraventricular (nontraumatic) haemorrhage, Rhesus and ABO isoimmunization, etc.
	A5	Disorders related to fetal growth	Small for gestational age, macrosomia, post-term, etc.
	A6	Antepartum death of unspecified cause	Intrauterine death of unspecified cause
**Intrapartum death**	I1	Congenital malformations and chromosomal abnormalities	Anencephaly, encephalocele, microcephaly, congenital hydrocephalus, spina bifida, etc.
	I2	Birth trauma	Intracranial laceration and haemorrhage due to birth injury, Fracture of skull due to birth injury, etc.
	I3	Acute intrapartum event	Intrauterine hypoxia
	I4	Infection	Congenital syphilis, congenital malaria, congenital rubella syndrome, congenital TB, etc.
	I5	Other specified intrapartum disorder	Vasa previa, ruptured cord, twin-twin transfusion, Intraventricular (nontraumatic) haemorrhage, Rhesus and ABO isoimmunization, etc.
	I6	Disorders related to fetal growth	Small for gestational age, extreme low birthweight, macrosomia, post-term, etc.
	I7	Intrapartum death of unspecified cause	Fetal death of unspecified cause
**Neonatal death**	N1	Congenital malformations, and chromosomal abnormalities	Anencephaly, encephalocele, microcephaly, congenital hydrocephalus, spina bifida, etc.
	N2	Disorders related to fetal growth	Small for gestational age, exceptionally large baby, post-term, etc.
	N3	Birth trauma	Cerebral haemorrhage due to birth injury, intraventricular haemorrhage due to birth injury, etc.
	N4	Complications of intrapartum events	Intrauterine hypoxia, birth asphyxia
	N5	Convulsions and disorders of cerebral status	Neonatal cerebral irritability, neonatal cerebral depression, neonatal coma, etc.
	N6	Infection	Tetanus neonatorum, bacterial meningitis, bacterial sepsis, congenital pneumonia, etc.
	N7	Respiratory and cardiovascular disorders	Respiratory distress syndrome, Neonatal aspiration syndromes, neonatal cardiac failure, neonatal cardiac dysrhythmia, neonatal hypertension, etc.
	N8	Other neonatal conditions	Vasa previa, ruptured cord, twin-twin transfusion, Rhesus and ABO isoimmunization, kernicterus, etc.
	N9	Low birthweight and prematurity	Extremely low birth weight, extreme immaturity
	N10	Miscellaneous	Cases where codes from several other sections of ICD-10 should be used
	N11	Neonatal death of unspecified cause	Congenital renal failure, termination of pregnancy, affecting fetus and newborn, withdrawal symptoms from drugs, etc.
**Maternal conditions**	M1	Complications of placenta, cord and membranes	Abruptio placentae, prolapsed cord, chorioamnionitis, etc.
	M2	Maternal complications of pregnancy	Premature rupture of membranes, oligo- and polyhydramnios, ectopic pregnancy, multiple pregnancy, etc.
	M3	Other complications of labour and delivery	Breech delivery and extraction, forceps delivery, Caesarean delivery
	M4	Maternal medical conditions	hypertensive disorders, maternal injury, maternal use of tobacco, alcohol or drugs, etc.
	M5	No maternal conditions	No condition identified

### Data analysis

Descriptive analyses were conducted using SPSS (IBM, NY, version 22). Rates were calculated using 95% confidence intervals. ANOVA, chi-square and t-test were used to compare population characteristics, and p-value of ≤ 0.05 was considered significant.

## Results

Overall, 1267 stillbirths were included: 321 in Kenya, 299 in Malawi, 340 in Sierra Leone and 307 in Zimbabwe. The hospital stillbirth rate varied among countries: facilities in Malawi had the lowest rate (20.3 per 1000 births, 95% CI: 15.0–42.8), followed by those in Zimbabwe (34.7 per 1000 births, 95% CI: 31.8–39.2), Kenya (38.8 per 1000 births, 95% CI: 33.9–43.3) and Sierra Leone (118.1 per 1000 births, 95% CI: 115.0–121.2).

### Characteristics

The mean age of the mothers was 26.2 years (SD 6.4), with only a slight variation between the countries (F (3, n = 1227) = 6.3, p = 0.0003; Table 2), with a small effect size of 0.02. The mean age of mothers varied by ante- or intrapartum death (p = 0.037), but no difference was observed in parity between the two groups (p = 0.236). The mean gestational age was estimated at 35.8 weeks (SD 3.5; ANOVA: F (3, n = 1194) = 11.16, p < 0.0005; effect size = 0.003). However, gestational age was largely estimated from last menstrual period; only 54 cases (4.3%) had an ultrasound scan in early pregnancy for confirmation of gestational age. Most babies (68.2%) were born via vaginal birth, while 303 (23.9%) were delivered by caesarean section, and 60 (4.7%) of the mothers had a laparotomy for ruptured uterus.

**Table 2:** Demographic and clinical characteristics of the study population.

Characteristics		Kenya n = 321 (%)	Malawi n = 299 (%)	Sierra Leone n = 340 (%)	Zimbabwe n = 307 (%)	Antepartum n = 532 (%)	Intrapartum n = 643 (%)	Total n = 1267 (%)	Statistic (Countries Comparison)
**Maternal age (years)**	< 15	0 (0.0)	4 (1.3)	1 (0.3)	0 (0.0)	2 (0.4)	2 (0.3)	**5 (0.4)**	
	15–19	29 (9.0)	47 (15.7)	81 (23.8)	34 (11.1)	69 (13.0)	108 (16.8)	**191 (15.1)**	
	20–24	91 (28.1)	93 (31.1)	77 (22.7)	75 (24.4)	146 (27.4)	164 (25.5)	**336 (26.5)**	
	25–29	101 (31.5)	63 (21.1)	80 (23.5)	79 (25.7)	132 (24.8)	168 (26.1)	**323 (25.5)**	
	30–34	51 (15.9)	47 (15.7)	49 (14.4)	63 (20.5)	89 (16.7)	106 (16.5)	**210 (16.6)**	
	35–39	34 (10.6)	27 (9.0)	34 (10.0)	35 (11.4)	61 (11.5)	62 (9.6)	**130 (10.3)**	
	> = 40	8 (2.5)	10 (3.3)	7 (2.1)	11 (3.6)	21 (3.9)	12 (1.9)	**36 (2.8)**	
	No information	7 (2.2)	8 (2.7)	11 (3.2)	10 (3.3)	12 (2.3)	21 (3.3)	**36 (2.8)**	
	Mean {SD}	26.6 {5.8}	25.7 {6.6}	25.2 {6.4}	27.2 {6.5}	26.6 {6.5}	25.8 {6.3}	**26.2 {6.4}**	
	ANOVA (age by country)	-	-	-	-	-	-	**-**	F = 6.3; p = 0.0003
**Parity**	Para 1	103 (32.1)	101 (33.8)	101 (29.7)	105 (34.2)	171 (32.1)	211 (32.8)	**410 (32.4)**	
	Para 2–4	195 (60.8)	162 (54.2)	172 (50.6)	193 (72.9)	311 (58.5)	362 (56.3)	**722 (57.0)**	
	Para 5 or more	16 (5.0)	31 (10.4)	60 (17.7)	8 (2.6)	41 (7.7)	59 (9.2)	**115 (9.1)**	
	ANOVA (parity by country)	-	-	-	-	-	-	**-**	F = 18.4; p = 0.0005
**Antenatal visit**	At least 1 visit	272 (84.7)	207 (69.2)	146 (42.9)	222 (72.3)	295 (55.5)	371 (57.7)	**847 (66.9)**	
	4 or more visits	113 (35.2)	29 (9.7)	4 (1.2)	75 (24.4)	101 (19.0)	112 (17.4)	**221 (17.4)**	
**Referral status**	Referral from other healthcare facility	118 (36.8)	99 (33.1)	139 (40.9)	213 (69.4)	247 (46.4)	267 (41.5)	**569 (44.9)**	
	Direct admission	198 (61.7)	191 (63.9)	196 (57.7)	88 (28.7)	274 (51.5)	364 (56.6)	**673 (53.1)**	
	No information	5 (1.6)	9 (3.0)	5 (1.5)	6 (2.0)	0 (0)	12 (1.9)	**25 (2.0)**	
**Type of pregnancy**	Singleton	294 (91.6)	269 (90.0)	302 (88.8)	284 (92.5)	494 (92.9)	576 (89.6)	**1,149 (90.7)**	
	Multiple	20 (6.2)	29 (9.7)	25 (7.4)	15 (4.9)	25 (4.7)	55 (8.6)	**89 (7.0)**	
	Chi-square (country)	-	-	-	-	-	-	**-**	*X*^*2*^ = 5.42; p = 0.14
**Gestational age at birth**	28 to 31 completed weeks	70 (21.8)	24 (8.0)	29 (8.5)	63 (20.5)	109 (20.5)	68 (10.6)	**186 (14.7)**	
	32 to 36 completed weeks	85 (26.5)	70 (23.4)	94 (27.6)	102 (33.2)	162 (30.5)	169 (26.3)	**351 (27.7)**	
	37 completed weeks or more	148 (46.1)	179 (59.9)	209 (61.5)	125 (40.7)	233 (43.8)	373 (58.0)	**661 (52.2)**	
	No information	18 (5.6)	26 (8.7)	8 (2.4)	17 (5.5)	28 (5.3)	33 (5.1)	**69 (5.5)**	
**Mode of delivery**	Vaginal delivery	230 (71.7)	192 (64.6)	227 (66.8)	218 (71.0)	408 (76.7)	397 (61.7)	**867 (68.4)**	
	Caesarean section	76 (23.7)	69 (23.1)	77 (22.6)	81 (26.4)	99 (18.6)	203 (31.6)	**303 (23.9)**	
	Laparotomy	8 (2.5)	23 (7.7)	22 (6.5)	7 (2.3)	13 (2.4)	22 (3.4)	**60 (4.7)**	
	Instrumental (assisted) vaginal delivery	1 (0.3)	9 (3.0)	8 (2.4)	0 (0.0)	4 (0.8)	14 (2.2)	**18 (1.4)**	
	Destructive operation (craniotomy)	0 (0.0)	4 (1.3)	0 (0.0)	0 (0.0)	2 (0.4)	2 (0.3)	**4 (0.3)**	
	No information	6 (1.9)	2 (0.7)	6 (1.8)	1 (0.3)	6 (1.2)	5 (0.8)	**15 (1.2)**	

### Time of death

Four hundred and fifty-five (455) births were recorded in case notes and registers as fresh stillbirths, accounting for 35.9% of all cases. The remainder were recorded as macerated (53.2%) or the physical appearance was not recorded at all (10.9%). One-fifth (21.1%) of all cases were macerated stillbirths whose fetal heart sound was documented as present on admission (Table 3).

**Table 3:** Time of death.

Fetal Heart Sound Documented	Fresh Stillbirths (n = 455)	Macerated Stillbirths (n = 674)	Unspecified (n = 138)	Total (n = 1267)
**Present**	226 (49.7)	142 (21.1%)	46 (33.3%)	414 (32.7%)
**Absent**	162 (35.6)	456 (67.7%)	61 (44.2%)	679 (53.6%)
**Not documented**	67 (14.7)	76 (11.3%)	31 (22.5%)	174 (13.7%)

**Fig 1:**
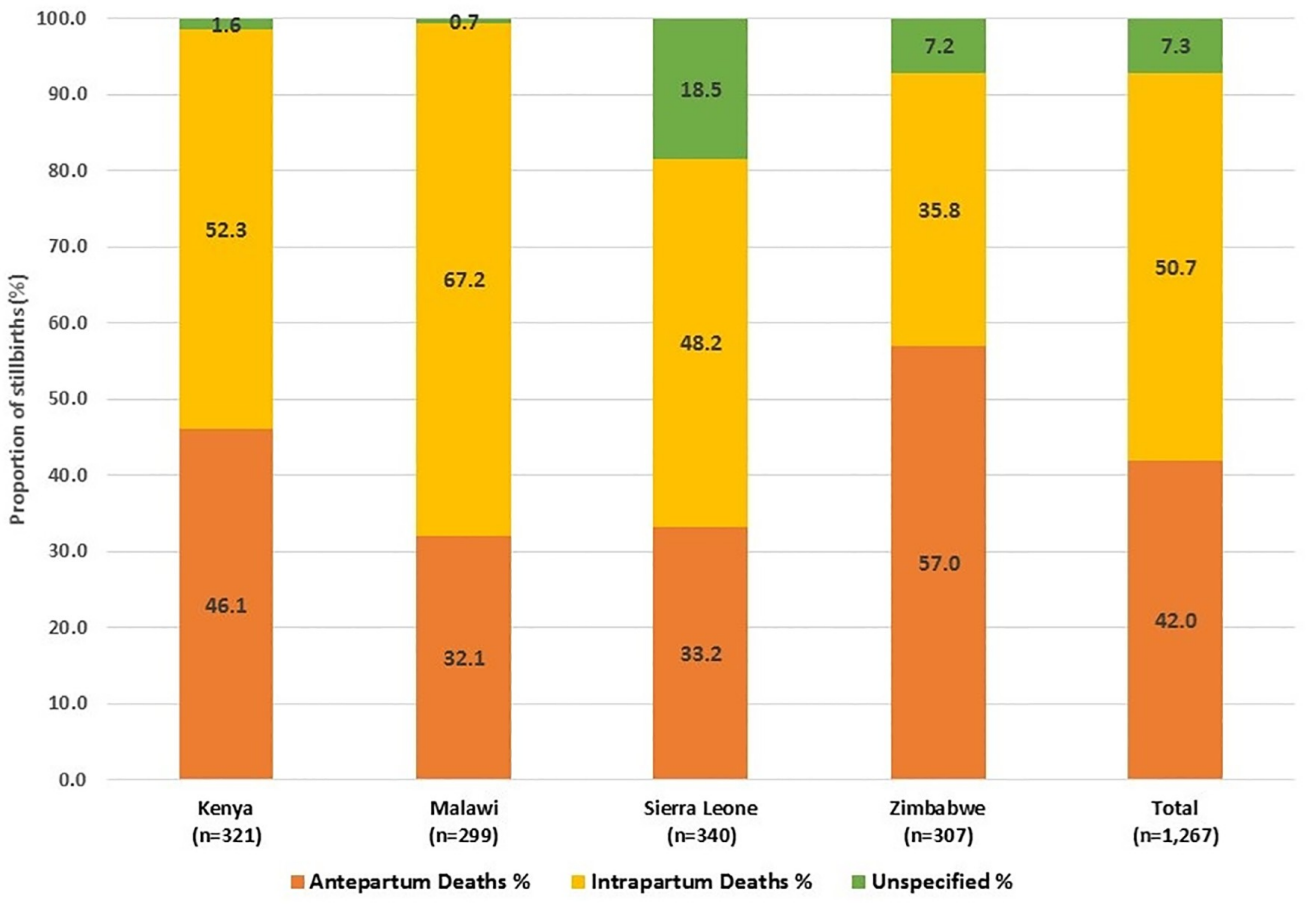
Distribution of stillbirths by timing of death.

For the purpose of the study, based on both physical appearance at birth and presence/absence of fetal heart sound on labour admission, of the 1,267 cases, 532 (42.0%) were considered antepartum deaths. The highest proportion of antepartum stillbirths was recorded in Zimbabwe. Intrapartum deaths constituted a total of 643 of the 1,267 cases (50.7%). Malawi had the highest proportion of intrapartum deaths, while Zimbabwe had the lowest. The remaining 92 cases (7.3%) could not be categorised either as antepartum or intrapartum deaths and were categorised as unknown time of death. Sierra Leone had the highest proportion of stillbirths that could not be categorised by time of death (Fig 1).

### Categories for cause of stillbirth

#### Antepartum deaths

Of the 532 deaths that were considered antepartum deaths, 207 cases (38.9%), all of which were macerated stillbirths, were classified into the category for other complications of labour and delivery (M3), which represents deaths where there is a contribution from breech delivery and extraction and Caesarean deliveries. These were removed from this part of the analysis, leaving a total of 325 antepartum deaths.

The majority (88.9%) were categorised as death of unspecified fetal cause. For stillbirths with a known cause, infections were the most frequent conditions identified (8.6% of 325), followed by the category for congenital anomalies, deformations and chromosomal abnormalities (2.2%) (Table 4).

**Table 4:** Application of ICD-PM to cause of stillbirth.

	M1 (Complications of placenta, cord and membranes)	M2 (Maternal complications of pregnancy)	M3 (Other complications of labour and delivery)	M4 (Maternal medical and surgical conditions)	M5 (No maternal condition)	Total (%)
**Antepartum Death**						
A1: Congenital malformations, deformations and chromosomal abnormalities	1	1	--	2	3	**7 (2.2)**
A2: Infection	4	2	--	22	0	**28 (8.6)**
A3: Antepartum hypoxia	0	0	--	0	0	**0 (0.0)**
A4: Other specified antepartum disorder	1	0	--	0	0	**1 (0.3)**
A5: Disorders related to fetal growth	0	0	--	0	0	**0 (0.0)**
A6: Antepartum death of unspecified cause	95	33	--	55	106	**289 (88.9)**
**Total n = 325 (%)**	**101 (31.1)**	**36 (11.1)**	**--**	**79 (24.3)**	**109 (33.5)**	**325 (100.0)**
**Intrapartum Death**						
I1: Congenital malformations, deformations and chromosomal abnormalities	2	2	10	1	9	**24 (3.7)**
I2: Birth trauma	0	0	0	0	0	**0 (0.0)**
I3: Acute intrapartum event	12	19	98	14	58	**201 (31.3)**
I4: Infection	1	5	5	12	0	**23 (3.6)**
I5: Other specified intrapartum disorder	0	0	0	1	1	**2 (0.3)**
I6: Disorders related to fetal growth	0	0	0	0	0	**0 (0.0)**
I7: Intrapartum death of unspecified cause	139	38	134	30	52	**393 (61.1)**
**Total n = 643 (%)**	**154 (24.0)**	**64 (10.0)**	**247 (38.4)**	**58 (9.0)**	**120 (18.7)**	**643 (100.0)**
**Stillbirth of Unknown Time of Death**						
U1: Congenital malformations, deformations and chromosomal abnormalities	0	0	2	0	0	**2**
U2: Hypoxia and other acute events	0	3	4	0	6	**13 (14.1)**
U3: Infection	1	0	0	1	0	**2 (2.2)**
U4: Other specified disorder	0	0	0	0	0	**0 (0.0)**
U5: Disorders related to fetal growth	0	0	0	0	0	**0 (0.0)**
U6: Death of unspecified cause	27	6	17	9	16	**75 (81.5)**
**Total n = 92 (%)**	**28 (30.4)**	**9 (9.8)**	**23 (25.0)**	**10 (10.9)**	**22 (23.9)**	**92 (100.0)**
**GRAND TOTAL—n = 1060 (%)**	**283 (26.7)**	**109 (10.3)**	**270 (25.5)**	**147 (13.9)**	**251 (23.7)**	**1060 (100.0)**

In contrast, for the majority (66.5%) of antepartum deaths could be classified into one of the groups for associated maternal condition. M1 (complications of placenta, cord and membranes) contributed the highest proportion (31.1%). Overall, 106 (32.6%) of the antepartum deaths had neither a fetal cause nor an associated maternal condition identified.

#### Intrapartum deaths

The major cause of intrapartum deaths, accounting for one-third (31.3%) of the 643 intrapartum deaths were acute intrapartum events (I3 category), mainly as a result of intrapartum asphyxia. The fetal cause of death for the majority (61.1%) of intrapartum deaths was unknown (Table 4).

As for contributing maternal conditions, 81.3% could be classified. M3 category (other complications of labour and delivery) was the most common category accounting for more than one-third of the intrapartum deaths. In contrast to antepartum stillbirths, only 52 (8.1%) of intrapartum stillbirths could not be assigned a fetal cause or an associated maternal condition.

#### Unknown time of death

Among the 92 cases whose time of death could not be determined, only 1 in 5 cases (18.5%) had a fetal cause identified. Hypoxia and other acute events, categorised as U2 in the study, was the most common fetal category assigned.

However, 76% of the cases could be classified by contributing maternal condition, an information that could have been lost if these cases were excluded because of unknown time of death. M1 (complications of placenta, cord and membranes) was the most common maternal condition assigned, accounting for 30.4% of the cases in this group. Only 16 (17.4%) of the cases with unknown time of death had neither fetal cause assigned nor maternal condition associated with the death identified.

## Discussion

### Main findings

In this study, the majority of stillbirths occurred intrapartum (51%) with 42% occurring antepartum.

The time of death for the remaining cases (7%) could not be determined. Overall, the proportion of stillbirths for which no fetal or maternal cause could be determined was much higher in antepartum deaths (32.6%) than for intrapartum deaths (8.1%).

The most commonly identified cause for antepartum deaths was infection (A2; 8.6% of antepartum stillbirths). Acute intrapartum events (I3) accounted for the largest proportion of intrapartum deaths (31.3%). No specific fetal cause of death could be determined in 9 out of 10 antepartum stillbirths and in 6 out of 10 intrapartum deaths. For associated maternal conditions, 76% of cases could be assigned a category. The M1 category (complications of placenta, cord and membranes) was the most common category assigned for antepartum deaths, while complications of labour and delivery (M3) accounted for the highest proportion of intrapartum deaths.

### Strengths and limitations

To our knowledge, this is the first and the largest study that has reported findings of the application of the new ICD-PM classification to identify and classify the causes of stillbirths which occurred in LMIC. It is hoped that the findings will inform considerations for future amendments to the ICD-PM classification system. A previous study by Allanson et al applied the ICD-PM to data which was obtained mainly from stillbirths which had occurred in the UK [9]. In our study, 92/1267 stillbirths could not be categorised as ante- or intrapartum death, but complete information loss from such cases was avoided by provisionally creating an additional category in ICD-PM. For 8 out of 10 cases with unspecified time of death, a clear cause of death could not be assigned but in 76% of cases there were contributing maternal conditions.

Lack of diagnostic capacity, incompleteness and inaccuracy of clinical records remain huge challenges in many LMIC. In this study, poor records presented a problem across all study countries. For example, gestational age was largely estimated from last menstrual period, which is not as reliable as using a first trimester scan [13]. However, whenever possible, information was obtained and triangulated from as many different sources as possible, including patient records, registers and hand-over notes.

### Interpretation of findings

The stillbirth rate was high across the four countries, and extremely high in Sierra Leone. This may be because the period of the data collection coincided with Ebola virus outbreak in the West African region, which affected maternal and newborn health services [14].

Determining time of fetal death is difficult, especially in LMIC where antenatal and intrapartum care is often sub-standard and fetal heart rate monitoring and/or growth monitoring is poor [15]. Furthermore, a study from Ghana that evaluated the reliability of provider assessment of fetal maceration by physical appearance found that 30% of fresh stillbirths were misclassified and reported as macerated. The authors concluded that provider-reported fetal appearance alone is an unreliable indicator for assessing time of fetal death [15].

In this study, a combination of both fetal appearance and the presence/absence of fetal heart sound on admission were used as criteria to determine if a stillbirth had occurred ante- or intrapartum. Consequently, 50.7% of all the stillbirths were classified as intrapartum. This confirms previous estimates by Lawn et al who combined global estimates suggesting up to 51% of all stillbirths are intrapartum stillbirths [1]. The variation observed between countries in the proportion of stillbirths classified as ante- or intrapartum death was likely due to variation in the capacity of the participating health facilities to deliver emergency obstetric care. Even though all the health facilities in the study were designated to provide high quality skilled birth attendance and comprehensive emergency obstetric care, their capacity to do so varied.

A minority of the cases in this study (7.3%) could not be categorised as ante- or intrapartum. Other studies have reported much higher proportions [16,17,18,19,20]. In a smaller study of 153 stillbirths in a hospital in East Timor, Wilkins et al. reported that the time of death for up to one-third (31.4%) of stillbirths could not be determined [16]. However, this study included all births ≥500 g or with gestational age of ≥22 weeks, which might have affected the proportions of stillbirths which would have been antepartum, intrapartum or unknown.

Visible signs of maceration begin to show 6–12 hours post-mortem [21]. Thus, in general, for mothers admitted in active labour with a live baby, a stillbirth outcome would be expected to be fresh rather than macerated. In this study, one-fifth (21.1%) of all cases were recorded as being macerated stillbirths with a fetal heart sound; documented as being present at the time of admission. It remains difficult to know whether the fetal heart rate was wrongly recorded as being present and/or whether the description of maceration was accurate. There are reports of misclassification by healthcare providers of a stillbirth as macerated when this is actually a fresh intrapartum stillbirth in order to avoid blame [15]. It is also important to note that the early signs of maceration are less likely to be recognised by untrained healthcare providers [15], increasing the risk of misclassification when relying on physical appearance alone.

Allanson et al reported 50% and 48% of stillbirths as antepartum in South Africa and the UK, respectively. They also reported 11% (South Africa) and 5% (the UK) of stillbirths as being intrapartum [9]. It is noteworthy, however, that they reported on perinatal deaths, i.e. including early neonatal deaths, which accounted for 39% of all cases in South Africa and 47% in the UK. The definition of stillbirth for data from the UK was also different i.e. including stillbirths from 24 weeks of gestation. For stillbirths alone, intrapartum deaths occurred in 17.7% and 9.4% of cases in South Africa and the UK, respectively, which is very much lower than our results (50.7%) and the estimated global figures for intrapartum stillbirths (51%) [1]. In another study that used South African data alone, Lavin et al found that of the 26,810 cases, 58.2% were antepartum, 14.4% were intrapartum and 27.8% were neonatal deaths [10]. The study also agrees with our finding that, using the ICD-PM, more cases had an associated maternal condition that fetal cause of death.

Since application of the ICD-PM classification currently requires that stillbirths are first classified by time of death (ante- or intrapartum) before a fetal cause of death and/or an associated maternal condition can be assigned, it will be challenging to apply the new ICD-PM classification to stillbirths from LMIC if there is a lack of information that allows accurate determination of time of death. At a minimum, a more accurate documentation of fetal appearance (fresh or macerated) should be in place together and regular fetal heart rate monitoring is vital. Thus, improvement in clinical care as well as documentation is needed to allow more accurate determination of the time and cause of death. Although we tried to apply ICD-PM for stillbirths with unknown time of death, this is not perfect. Nevertheless, it does provide additional information about the cases upon which action can be based.

In the short term, therefore, the introduction of an additional category in the ICD-PM classification system for stillbirths with unknown time of death would be useful.

#### Cause of death

Generally, far more associated maternal conditions were identified than fetal causes of death. This may be because maternal complications largely rely on clinical diagnosis and can be more easily identified and recognised by healthcare providers. These included complications of placenta, cord and membranes.

We found that “disorders related to fetal growth” (A5 and I6) were particularly difficult to assign. This was mostly due to the difficulty in assessing intrauterine growth retardation.

In their study, Allanson et al. found 59.1% and 22.4% of antepartum and intrapartum deaths, respectively, to be due to an unspecified fetal cause [9]. Furthermore, 53.3% of the antepartum deaths had no identified associated maternal condition. Thus, for intrapartum death, Allanson et al reported a comparatively smaller proportion with unspecified fetal cause (22.4%), and a larger proportion without associated maternal conditions (37.9%) [9]. In contrast, in LMIC, obstetric complications still account for most of the intrapartum stillbirths [3,4].

The structure of the ICD-PM categories may also require further revision to avoid misclassification. For example, it is difficult to associate antepartum deaths with “other complications of labour and delivery” (M3) since an antepartum death will by definition have occurred before labour and therefore have little to do with events during labour or birth. New guidelines for the application of the ICD-PM should highlight this and other potential pitfalls. It should be noted, however, that some of the challenges of the ICD-PM identified in this study are inherent in the ICD-10. It is important that this considered in the development of ICD-11.

## Conclusion

Healthcare providers in LMIC should be aware of the need and supported to improve clinical care and documentation of care for all mothers, including those who experience stillbirth as well as the stillborn babies. Diagnostic protocols and guidelines for the application of these need to be introduced. In the interim, it is recommended that an additional category is added to the ICD-PM to accommodate stillbirths with unknown time of death.

## Supporting information

S1 File Data collection tool. (PDF)
