# Supplementary material for: Application of the ICD-PM classification system to stillbirth in four sub-Saharan African countries
Source: PLoS One. 2019 May 9;14(5):e0215864. doi: 10.1371/journal.pone.0215864 (PMC6508706; doi:10.1371/journal.pone.0215864)
Supplement: S1 File — (PDF) [file pone.0215864.s001.pdf]

PERINATAL DEATH REVIEW FORM

Instructions: Fill in this form for every perinatal death (i.e. stillbirth and neonatal death in the 1st week of life). The example of code below (SRL/FRT/01/01) represents "Sierra Leone/Freetown/January/1st case of perinatal death in the month of January".

**Country**	**Date of Audit**	**Date of Data Collection**	**Code (E.g. SRL/FRT/01/01)**

SECTION 1: HEALTH FACILITY

Name of Health Facility: ……………………………………………………………………………………………………

County / District / Region: ……………………………………………………………………………………..................

Type of Health Facility (tick one):

National	Referral / Teaching	Regional	District	General	Health Centre	Other (specify) …………

Level of Health Facility (tick one): BEmONC ☐ CEmONC ☐

Is this a Helping Baby Breath (HBB) Facility? Yes ☐ No ☐

SECTION 2: MOTHER

Mother's Initials: ………………………………………… **Mother's Age:** .... (in years)

Mother's Address (tick one): Rural☐ Semi-urban☐ Urban☐

Mother's County / District: ………………………………………………………………………………………………….

Educational Level Completed (tick one):

None ☐

Primary ☐

Secondary ☐

Tertiary ☐

Was mother referred from another facility? Yes ☐ No ☐

If Yes, from which facility? …………………………………………………………………………………………………..

SECTION 3: PREGNANCY

Mother's Parity: Para ……………….…. + …………………. No. of Children Alive ……………………..

Type of pregnancy (tick one): Singleton☐ Multiple Gestation☐

Antenatal Care Attendance: Yes ☐ No ☐

If yes, how many visits? ……………………………….

Date of 1st Booking: …………………………………….

ANC Interventions (tick all that apply):

Interventions	Yes	No	Follow-Up Questions	Response to Follow-Up Questions
Iron and Folate given?			If yes, for how long?	
Anti-malaria prophylaxis given?			If yes, how many doses?	
Tetanus Toxoid given?			If yes, how many doses?	
HIV test done?			If yes, +ve or –ve result?<br>If +ve, on ARV?	
Syphilis test done?			If yes, +ve or –ve result?<br>If +ve, any treatment?	
Rhesus blood group checked?			If yes, +ve or –ve result?<br>If RhD –ve, treatment?	

Conditions present during this pregnancy (tick all that apply)

Conditions	Yes	No	Follow-Up Questions	Response to Follow-Up Questions
Antepartum haemorrhage			If yes, mention treatment	
Malaria			If yes, mention treatment	
Hypertensive disorders (PIH, pre-eclampsia, eclampsia)			If yes, mention treatment	
Diabetes			If yes, mention treatment	
Pre-mature rupture of membranes			If yes, mention treatment	
Anaemia (Hb)			If yes, mention treatment	
Urinary tract infection			If yes, mention treatment	
Trauma (due to accident or gender-based violence)			If yes, mention treatment	
Other (specify)			If yes, mention treatment	

SECTION 4: LABOUR AND BIRTH

Gestational Age (in weeks): ………………….. **Cervical Dilatation on Admission:** …………. cm

Reason for Admission: ……………………………………………………………………………………………………

Date of Admission (DD/MM/YYYY): ……………………….… **Time of Admission**: …………………

Date of Delivery (DD/MM/YYYY): ……………………………. **Time of Delivery**: …………………….

Date of Discharge (DD/MM/YYYY): …………………………..

Place of delivery (tick one):

Health facility ☐ (specify) ……………………………………………………………………………….

Home ☐

TBA ☐

Other ☐ (specify) ………………………………………………………………………………..

On admission, was foetal sound present? Yes ☐ No ☐ Not assessed ☐

Was partograph used? Yes ☐ No ☐ Unknown ☐

If 'Yes', was partograph used correctly? Yes ☐ No ☐

If 'No', mention error: ……………………………………………………………………………………………….

Any Obstetric Complications? ……………………………………………………………………………………………

……

Presentation (tick one)**:**

Cephalic ☐

Breech ☐

Others (specify): ……………………………………………………………………………………………………..

Mode of Delivery (tick one)**:**

Spontaneous Vaginal Delivery ☐

Caesarean Section ☐

Vacuum ☐

Forceps ☐

Others (specify): ....

Indication(s) for Instrumental / Caesarean Delivery: ....

……

Time between decisions for CS / instrumental and actual delivery of the baby:

Less than 30 minutes ☐ 30 minutes - 1 hour ☐

Greater than 1 hour ☐ How Long? ………………. Not documented ☐

Mother's Outcome: Alive ☐ Died ☐

Any Morbidity? (Specify) ……………………………………………………………………………………….

SECTION 5: BABY'S CONDITION

Weight of the baby (in grams): ……………………………….

Sex: Female ☐ Male ☐

Baby's Condition at Birth: Alive ☐ Fresh Stillbirth ☐ Macerated Stillbirth ☐

Any congenital anomaly noted: ……………………………………………………………………………….

Apgar Score: At 1 minute: ………… At 5 minutes: …………. At 10 minutes: …………….

Resuscitation attempted with Ambu bag? Yes ☐ No ☐

If born alive, select one: Kept with Mother ☐ Referred ☐

If born alive, state when the baby died: ………………………………. (days or hours after delivery)

SECTION 6: CAUSES AND AVOIDABLE FACTORS

Probable Cause(s) of Death:

(E.g. congenital anomaly, HIV, hypertension, placenta previa, asphyxia, umbilical prolapse, ruptured uterus, etc.). If more than one cause, list the most likely cause(s) first.

1. ……
2. ……
3. ……
4. ……

Avoidable Factors: Use comment section for clarifications.

Factors	Yes	No	Support with facts
Delay to seek health care			
Delay to reach the health facility			
Delay to provide care after arrival at health facility			
Was full complement of staff available during mother's stay?			
Functional resuscitation equipment (e.g. ambu bag) available?			
Supplies and drugs (including blood) available?			
Were instructions, guidelines and/or protocols followed?			
Was the right intervention used?			
Was relevant and adequate documentation made?			
Others (specify):			

SECTION 7: PLAN OF ACTION

ACTION POINT	PERSON RESPONSIBLE	TIMEFRAME

Comments:

………

………

………

………

………

………

………

………

………

………

………

………

………

Form Completed by:

Name: ……………………………………………………………………… Sign: …………………… Date: ………………………...
